# Efficient integration of personal factors into the international classification of functioning, disability, and health (ICF): the importance of emotional and motivational aspects in goal pursuit

**DOI:** 10.3389/fresc.2024.1450157

**Published:** 2024-11-29

**Authors:** Ayano Tsuda, Emmanuel Manalo, Ichiro Miyai, Tomoyuki Noda

**Affiliations:** ^1^ Department of Brain Robot Interface, ATR Computational Neuroscience Laboratories, Kyoto, Japan; ^2^ Graduate School of Education, Kyoto University, Kyoto, Japan; ^3^ Neurorehabilitation Research Institute, Morinomiya Hospital, Osaka, Japan

**Keywords:** goal-setting, international classification of functioning, disability, and health (ICF), personal factors, motivation, self-efficacy, personality systems interaction (PSI) theory, goal-oriented action linking (GOAL) architecture

## Abstract

The International Classification of Functioning, Disability, and Health (ICF) is a widely used framework in rehabilitation that provides standardized measures to describe health and health-related states of people. The strength of the ICF lies in its provision of a common language for describing rehabilitation progress. However, personal factors are not classified within the ICF due to their significant variability across cultures, which may render it not adequately capturing the subjective and social dimensions of disability. Our objective in this research was to propose theoretical frameworks that could help identify relevant personal factors for inclusion in the ICF. We discuss the Personality Systems Interaction (PSI) Theory to identify personal variability in goal pursuit, highlighting the importance of emotions like negative and positive affect in handling adverse situations and managing habitual behaviors. Additionally, the theory helps to determine personality factors relevant to patients, facilitating the resolution of potential issues that may emerge during the goal achievement process. We also emphasize the role of goal setting in rehabilitation and suggest the Goal-Oriented Action Linking (GOAL) model as a useful tool for understanding how motivational values change over time, distance, and progress. Following from this, we discuss the importance of self-efficacy and its relationship to effort and goal achievement, while noting potential issues in its assessment. Finally, we propose viable assessment methods for measuring the potential components to be incorporated as personal factors.

## ICF and personal factors in stroke rehabilitation

Stroke is the leading cause of disability worldwide (1). This fact underscores the need for rehabilitation to support those who have suffered from a stroke. For stroke patients, it is important to formulate coordinated care from a large team to work towards achieving patient goals (2). Patient-centeredness is recognized as a key element of goal-setting (3, 4), though research with inpatient and early outpatient stroke patients suggests that goals may be confounded with hopes and dreams, which are often less specific and measurable (5). To promote better goal-setting practices, evidence-based healthcare is necessary (6, 7), which may be achieved in conjunction with the International Classification of Functioning, Disability and Health (ICF) to identify and structure patient goals (8–10).

The ICF is a multipurpose classification framework developed by the World Health Organization (WHO). The ICF is particularly beneficial in cases requiring multidisciplinary collaboration, and it has been demonstrated to facilitate communication among members of rehabilitation teams (11). Despite this strength, one aspect of ICF that has long been discussed as needing improvement is the aspect of “personal factors,” a subcategory of “contextual factors.” Personal factors are not classified in the current ICF because they are thought to vary considerably across societies and cultures (12), which leads to a lack of consensus about what aspects of personal factors should be included in the ICF (13, 14).

The objective of this paper is to propose theoretical frameworks from the field of psychology that could be considered as personal factors in the ICF. We will summarize the current knowledge on personal factors and put forward theoretical frameworks grounded in psychological theories to guide the selection of personal factors that could usefully be included in the ICF. Given the significant overlap between these psychological factors and the personal factors identified as important for inclusion in the ICF, we have decided to conduct a conceptual literature review to explore relevant theories with the aim of proposing potential approaches for rehabilitation practitioners to support patients in achieving their goals. These approaches will draw on theoretical frameworks from various psychological domains, primarily developed through studies with non-clinical participants, to establish criteria for identifying essential personal factors in the rehabilitation process, with the aim of exploring theoretical connections and proposing a conceptual framework. Therefore, a conceptual literature review was chosen to allow a more flexible and narrative exploration of the connections between psychological and rehabilitation theories rather than to systematically evaluate specific studies or interventions. This review focuses exclusively on stroke patients, given stroke’s prominence in rehabilitation, and seeks to apply psychological research findings specifically to this population. Stroke, as a topic of focus, facilitates a meaningful comparison with psychology for its involvement in brain control, motor, and cognitive learning processes, distinguishing it from rehabilitation for other conditions like fractures (15).

### The international classification of functioning, disability and health (ICF)

The aim of ICF is “to provide a unified and standard language and framework for the description of health and health-related states in order to improve communications between different users” (p. 3) (12). It uses a set of coding system for assessing two main aspects: functioning and disability, and contextual factors. There are four components within these two categories:

1. Functioning and disability
  a. Body function and structures: refer to changes in body functions in physiological and psychological terms, and structures that refer to anatomical changes in the components of the body.
  b. Activities and participation: refer to active engagement in activities, and participation pertains to involvement in life situations.
2. Contextual Factors
  a. Environmental: external factors that can either hinder or facilitate physical, social, and personal attributes.
  b. Personal factors: include aspects such as gender, age, race, lifestyle, and coping habits. Personal factors are not formally classified in the current version of the ICF because of social/cultural variations associated with it, but users can choose to identify them.

The WHO (12) proposes that the ICF may be used as a statistical, research, clinical, social policy and educational tool. The ICF provides aspects of how one can view health and functioning when providing specific services (16), and the use of the ICF in health-care goal-setting provides clinicians and patients with specific steps to follow when setting goals collaboratively (17).

Goal setting is considered to be an essential part of rehabilitation as it helps guide patients through the rehabilitation program by providing a framework for enhancing physical independence and psychological well-being (18). A crucial point about goal setting is that it needs to be tailored according to individual patient needs and to incorporate the necessary flexibility to accommodate the skills of the practitioners who will be implementing the corresponding practices. But the practitioners who provide healthcare, especially for complex medical conditions such as stroke and other forms of serious brain injury, usually have to work as part of a multidisciplinary care team with diverse expertise (19). Hence, goal setting needs to be structured around a more concrete process that would enable firm communication and coordination among the pertinent members of a team (2).

To tailor goals to individual needs and incorporate flexibility into the process it is necessary to provide practitioners with the skills and confidence to implement these practices (20) by using clinical guidelines, educating members of the multidisciplinary rehabilitation team about these guidelines, and evaluating practice and outcomes using comprehensive process indicators to improve quality of care in rehabilitation hospitals (21). In these situations, the ICF may be used to clarify the roles of the team members involved in the rehabilitation to aid communication and facilitate service provision (11). The ICF may also be used in providing feedback and setting goals with the individual, though agreed standards and evaluation are still lacking (22).

In situations requiring multidisciplinary care, the ICF can be effectively utilized to assess, plan and monitor the progress of rehabilitation. The advantage of the ICF is that it focuses not only on the bodily aspects of the individual but also on the environmental contextual factors that might contribute to their performance (12). The ICF defines disability as a contextual health experience arising from the interaction of health conditions and contextual factors, promoting a bio-psychosocial model that considers various influences on functioning, and emphasizes that disability is a universal phenomenon not determined by the nature of one’s health condition (12).

### Personal factors in the ICF

Despite the strengths of ICF in facilitating multidisciplinary care and considering the individual’s environment rather than solely focusing on body functions, the aspect of personal factors within the ICF has long been discussed as needing improvement. These are not classified in the ICF because they are thought to have considerable variation across cultures, lack of clarity in scope (12), and concerns exist about the possibility of putting on the responsibility of the disability on the individual (14, 23). Currently, it is up to rehabilitation practitioners to decide whether to consider personal factors when assessing individuals and providing rehabilitation.

Since personal factors and their relationship to other ICF categories can provide valuable insights into the selection of rehabilitation strategies and support (13, 14, 24), the information derived from personal factors should be considered together with other information on functioning and disability during the rehabilitation process (13, 24). However, there is a lack of a standardized and comprehensive understanding of which personal factors are of interest within the ICF framework, what falls beyond its scope, and how these factors should be distinguished from other ICF categories (13). Overlap between personal factors and the lack of definition and purpose for inclusion in ICF has also been pointed out as problematic (23).

To understand the conceptual use of personal factors as a starting point for formulating standardized measures and the kinds of categories of personal factors that exist, Geyh and colleagues (13) systematically reviewed and content analyzed how personal factors were addressed in research. They analyzed 79 articles, categorizing entities based on whether they included examples mentioned in the ICF, such as gender, social background, education, profession, past and current experiences, or content not explicitly mentioned in the ICF. They found 238 examples referred to as personal factors in 23 papers, which are not mentioned as personal factors in the ICF, with the most common being self-efficacy, followed by attitudes, expectations, and motivation. Similarly, Müller and Geyh (25) explored common ground in the categorization of personal factors, identifying categories such as emotional factors, personality, and motives/motivation, amongst others.

Since these were not based on guiding principles of the ICF, Geyh and colleagues (14) created a structural representation of the personal factors based on the guidelines of the ICF, which included aspects such as applicability and validity across cultures world-wide, and across a variety of health conditions. They identified three parts: individual facts, subjective experience, and recurrent patterns. The individual facts included information about their [1] socio-demographical factors, [2] position in the immediate social and physical context, and [3] personal history and biography. The subjective experience component included areas of [4] feelings, [5] thoughts and beliefs, and [6] motives. Lastly, recurrent patterns included [7] general patterns of experience and behavior, including categories such as patterns of thoughts and handling thoughts, patterns of behaviors and handling behaviors. They noted that formulation of these personal factors involves subjective perspectives, and other research findings differ in structure, highlighting the need for consensus. Additionally, they concluded that the structural representation created requires further consideration regarding its applicability to cultural, interdisciplinary, and multipurpose contexts.

We propose that in order to create a consensus among the different proposals for categorizations of personal factors, and to address the issue of cultural and interdisciplinary applicability, it is necessary to base the formulation of these categories on sound theoretical understanding of human cognition and behavior. The idea of “human universals” “traits or behaviors that are consistent across all people” is a fundamental assumption in psychology (26). Accordingly, we will present psychology-related theories to address this concept. Despite progress in understanding how culture evolves and varies, there is no clear agreement on cognitive universals (27). However, we believe that understanding the basis and mechanisms of human cognition and behavior will contribute to a “standardized representation and description of the lived experience of health from a personal factors perspective” (p. 1729) (14), while also raising important questions that warrant further investigation in future studies. As goals are crucial in rehabilitation (17, 28), and goals need to be clearly defined beforehand in order to match the individual’s needs (29) we will explain theories related to goal setting to identify essential individual traits that should be included as personal factors in the ICF.

In the following section, we will summarize theories and models related to goal-setting to elucidate the processes involved and identify relevant personal factors in the context of goals. We will also explore potential categorizations of personal factors for integration into the ICF framework and how various rehabilitation approaches align with these theories. We will propose specific strategies that rehabilitation practitioners can use in conjunction with the ICF.

## Psychological theoretical frameworks to be considered in personal factors

The objective of this section is to explain theoretical frameworks that have been proposed in the field of psychology to understand and explain motivation and goals. The Personality Systems Interaction Theory (PSI) (30) will be used in addressing the personal factors that may be important to categorize in the ICF. Additionally, Goal-Oriented Action Linking (GOAL) architecture (31), a model explaining the mechanisms behind how motivation of goal pursuit change depending on the type of goal (approach vs. avoidance), will also be explained to clarify the factors that are necessary to take into account when including them in the ICF as personal factors.

The inclusion of motivation as a distinct domain under personal factors is debatable, as it is already classified under body functions and structures with the following definition: “Mental functions that produce the incentive to act; the conscious or unconscious driving force for action” [ICF-code: b1301 (12)]. To determine if motivation should be distinctly categorized under personal factors, we will examine psychological theories relating to motivation. This examination will help to clarify the mechanisms behind motivation and identify aspects that are crucial for motivation to be categorized under personal factors.

The type of goals referred to in this review will use the theory of self-regulated learning (SRL) proposed by Carver and Scheier (32). They described two types of feedback loops, which may be used to understand the role of motivation. In negative feedback loops, which are also known as approach goals, the function is to reduce the discrepancy between people’s current status and their goal. In contrast, positive feedback loops, which are also known as avoidance goals, use “anti-goal” as a reference value and the objective of the loop is to move away from the goal. Approach and avoidance goals may operate in tandem, complementing each other in the pursuit of a desired outcome. For instance, in the context of physical fitness, one might adopt an approach goal, such as engaging in regular exercise, alongside an avoidance goal, such as limiting excessive food intake (32). Research has consistently found that individuals experience and approach the same task differently depending on whether their goal is framed as an approach or an avoidance goal (33). Even though SRL has been proposed as a valuable model for rehabilitation research (18), and goal-setting interventions based on SRL have been applied to patients with traumatic brain-injury (34), spinal cord injury, and stroke (35), we will initially focus on presenting the basic principles of PSI and the GOAL model, as these frameworks, to our knowledge, have not yet been empirically tested with stroke patients. Nonetheless, potential connections between these theories and existing findings related to stroke patients will be explored in the subsequent sections.

### The personality systems interaction theory (PSI)

**Figure 1:**
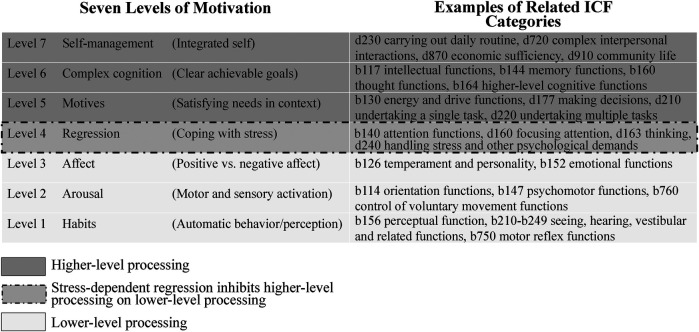
Aligning the seven levels of motivation with relevant ICF categories. Level of motivation adapted from Kuhl et al. (30).

The PSI was created to provide a unified definition of motivation and explain the personal variability involved in motivational pursuits (30). The PSI is important as it helps explain why behavior tends to change depending on the situation and time despite personality traits being stable (36, 37). According to the PSI theory, there are seven levels of motivation interconnected with theories of personality, which are shown in Figure 1. The bottom three levels are “lower-level processes,” characterized by minimal behavioral flexibility and automatic activation based on inputted information. In contrast, the top three levels are considered “higher-level processes,” which are more complex and interact with the lower processes to enhance behavioral flexibility. The comparison between the lower and higher-level processes occurs at level 4, and determines ongoing behavior. Level 4, known as *regression* is important as it relates to stress and explains why a person fails to sustain motivation in adverse situations. In Figure 1, we have also provided examples of possible ICF categories that may be linked to the seven levels of motivation based on the linking rule proposed by Cieza and colleagues (38).

The PSI theory posits that there is an interaction between the seven levels of motivational systems to understand how people integrate negative and positive experiences into their sense of self growth (30). Level 4 is accountable in comparing low-level inputs with higher-level systems to decide whether top-down or bottom-up processes should be used to determine the ongoing behavior. This can be achieved through two self-management competencies: first modulation assumption and second modulation assumption. The first modulation assumption, known as volitional efficiency (39) or action control (30), involves flexible switching between the top-level *intention memory* (level 7) and the low-level *intuitive behavior* (level 1), facilitated by positive affect. The second modulation assumption, also known as self-growth, involves flexible switching between *object recognition* and *extension memory* to learn from failures. Object recognition (level 1) identifies discrepancies between the current situation and a person’s expectations (40), using perceptual cues to detect errors or threats (30). This process is triggered by negative affect, signaling potential danger. In response, extension memory is activated to retrieve relevant information from past experiences, values, and needs (40, 41), offering strategies to address the problem (30). Given the relevance of the first modulation assumption to behavioral aspects in rehabilitation, the following section will focus on a detailed explanation of this assumption.

#### First modulation assumption

The important component of the first modulation assumption is the role of positive affect. Rather than acting on impulsive decisions, positive affect enables alignment of the person’s intention to their actual intended behavior. Whereas intuitive behavior enables execution of innate and learned behavioral routines, like habits, when difficulties arise, positive affect decreases and intention memory is activated. This switch inhibits intuitive behavior and activates intention memory so the person can analyze and plan for the necessary behavior (40).

Experimental evidence of the first modulation assumption is provided by using the Stroop tasks (42), which requires the person being tested to hold onto a challenging intention, such as focusing on the actual visual/ink color of the words shown (e.g., the red ink color of a word like “blue”). They must resist the strong habitual response of reading the word itself (“blue”), regardless of the color of the ink. In the context of PSI, intention memory tries to direct responses based on a deliberate goal (e.g., responding with “red”), while the intuitive behavior control system has a dominant automatic response tendency (e.g., responding with “blue”). Research with high school students has shown that when positive words like “success” or “excellent performance” are used as primes before a task involving incongruent color words, the interference usually caused by these incongruent words is significantly reduced or eliminated (42). This supports the idea that positive affect can facilitate the intended response over the automatic response. In context of rehabilitation practices, it might be beneficial to provide positive feedback to patients to reduce their tendency to rely on habitual responses. Feedback is known to be an important aspect as it enhances motor skill acquisition (43), and serves as a basis for error correction to achieve better performance for upcoming trials (44). However, the kinds of feedback to provide, the timing and how often feedback should be provided are debatable (44, 45), and determining those is a work in progress where stroke and other neurological diseases are concerned (46).

An important individual difference that accounts for the first modulation assumption is the person’s ability to control actions, which is classified as either action- or state-oriented. According to the action control theory (47, 48), which explains how individuals translate intention into action (49), action-orientation involves managing demanding situations by activating a change-promoting mode of control, while state-orientation refers to a change-preventing mode of control, where the current mental and behavioral state is sustained (50). When they experience failure, action-oriented individuals are able to manage negative emotions to achieve the goal (48), whereas state-oriented individuals ruminate over undesirable emotional states (51, 52). When the task demands are low and experienced emotions are positive, state-oriented individuals perform well, but when the tasks demands are high, action-oriented individuals tend to perform better (53).

Research has demonstrated that state-oriented individuals may learn self-regulation strategies. For example, one-time application of mental contrasting, an intervention that helps connect positive affect to an existing intention, facilitated goal achievement for state-oriented individuals but not for action-oriented individuals (51). Action-oriented individuals did not benefit from the intervention because they already used those self-regulatory strategies spontaneously. This highlights the importance of identifying these traits as personal factors, because inappropriate interventions may interfere with their goal pursuit. For stroke patients, interventions and training that promote self-management through methods such as goal-setting and self-monitoring have been shown to enhance quality of life and increase perceived ability to achieve goals (54). Therefore, self-management interventions may also be an important factor to consider.

### The PSI and potential personal factors to consider in the ICF

When considering personal factors to include in the ICF, it would be crucial to understand the valence of the patient’s emotions (either negative or positive), and whether they are state- or action-oriented individual. However, since the assumptions of the PSI are based on non-clinical settings, it is important to consider other ICF classifications and the patient’s medical history when applying these personality factors to stroke patients. Nonetheless, the advantage of utilizing the PSI framework lies in its ability to inform rehabilitation teams about personality factors relevant to their patients, aiding in the identification and resolution of potential issues that may arise in goal achievement. For example, Stroop tasks can help trace the pathway from intention memory to intuitive behavioral control and, when used alongside sensitivity to positive word priming, assess an individual’s ability to resist habitual responses. PSI may be of strong relevance in the ICF, as it provides a mechanism for describing current and expected levels of functioning and helps in formulating appropriate treatment plans and interventions.

In the representational structure for personal factors created by Geyh and colleagues (14), their classification under subjective experience includes *feelings*, *thoughts and beliefs*, and *motives*, which are also considered important factors in the PSI. Of utmost importance, the classification of *feelings* could be categorized in a valence of negative and positive to better capture the situation the person is dealing with. In other words, the emotional valence should be considered in relation to how capable the person is when facing adverse situations, such as failures, and how much they can control their habitual behaviors. The person’s ability to control their behavior, in terms of state- or action-orientation may be another aspect that is of importance, as it provides information on how people manage in adverse situations and whether they are susceptible to external influences.

In addition, the category of *recurrent patterns* in Geyh and colleagues’ (14) representational structure, which includes items related to general patterns of experience and behavior, including habits, may also be considered in relation to the seven levels of motivation. For instance, if an individual tends to rely on habits during rehabilitation, identifying those habits using the ICF could provide valuable insights into managing these routines when patients experience negative emotions, helping them avoid resorting only to familiar routines when difficulties arise.

To distinguish motives classified under body functions and structures, we propose that the *motives*, if classified under personal factors, may refer more to the definition used in the PSI, as higher-level processes that control or inhibit lower-level habits and emotional impulses so that goals may be met (30). In such cases, the role of negative vs. positive affect may be the central component. Positive affect facilitates flexible switching between lower- and higher-level functioning as in the first modulation assumption, and negative affect activates object recognition to inform about threats in a situation and use extension memory to retrieve information from past experiences to deal with the issue at hand, as in the second modulation assumption. Whereas the motives in body functions and structures emphasize mental functions, the definition used in personal factors may be focused more on the aspect of *feelings*.

The measurements used in the PSI, such as the Stroop task, are also used in rehabilitation research, but with different purposes. For instance, auditory Stroop tasks are commonly used with stroke patients as a cognitive tasks in dual-task situations to limit the attentional and switching task capacity of individuals when walking and crossing obstacles (55, 56), and turning-while-walking (57). Stroop tasks have been used in dual-task interference in conjunction with laboratory and real-world settings (58). Additionally, Dahdah and colleagues (59) explored whether immersive Virtual Reality (VR) interventions improve executive dysfunction in brain injury patients and compared performance on a VR Stroop task with traditional formats (ANAM Stroop, Delis-Kaplan Executive Function Stroop, Golden Stroop, and WJ-III: Pair Cancellation). Results indicated that VR Stroop tasks with distracters (auditory, visual, audiovisual) were more challenging than traditional tasks. While patients responded faster in VR, they also displayed more impulsive behaviors and errors, highlighting difficulties in such environments. This suggests that VR Stroop tasks may better identify cognitive deficits in these patients.

There are different versions of the Stroop tasks available and their reliability has been questioned (60) due to the tasks’ complex multifactorial structure (61). For instance, taking into consideration Golden’s Stroop task (62), Periáñez and colleagues (61) found that the Stroop word-reading (colors printed in black ink) reflected speed of visual search, the Stroop color-naming (item XXXXX printed in color) reflected working memory and speed of visual search, and the Stroop color-word (aka. interference, in which color and word do not match) reflected working memory, conflict monitoring, and speed of visual search. If this is the case, because post-stroke patients are also known to have working memory deficits (63), it would be necessary to use Stroop tasks in conjunction with other ICF categories under functioning and disability for assessment purposes. This approach will help identify potential deficits that patients may have when using the Stroop task and if such deficits are identified, alternative assessment methods should be employed.

### Factors influencing goal pursuit

Up to now, we have focused on individual differences related to sustaining motivation. An additional critical consideration in identifying relevant personal factors is the attractiveness of goals and the associated motivational factors influencing goal pursuit. Goals that are not perceived as attractive by individuals are less likely to be pursued. Since personal factors related to individual tendencies may vary in terms of perceived value, in the next section, we will examine these possible influencing factors, including the perception of one’s own ability.

#### Goal setting, self-efficacy, and role of motivation

Goal setting has been demonstrated to enhance performance (64, 65), and there is a reciprocal relationship between self-efficacy beliefs and goals (66). According to Bandura (65, 67, 68), self-efficacy pertains to individuals’ assessments of their abilities to perform particular tasks. Individuals who perceive themselves as capable are more likely to set higher goals and demonstrate stronger commitment to achieving them (69), and progressive attainment of goals enable them to sustain their sense of self-efficacy (66).

Goal setting is particularly advantageous for individuals with low self-efficacy, highlighting the importance of assisting them in establishing attainable goals and providing guidance on the appropriate timing for goal-related performance (70). A qualitative interview study with stroke survivors found that gradual increases in their skills and engagement in daily activities were related to the development of their sense of confidence (71). In such cases, goal setting requires the goals to be proximal and achievable in the short-term (64, 72, 73). That is, the more the goal is closely connected to the individual’s current attainment level, the more likely it is that the goal will be achieved (64, 72, 74), although there are some studies that suggest distant and more challenging goals are necessary for promoting better performance (75). From this perspective, Schunk (72) suggested that the important factor is to subdivide the distal goals into achievable goals by understanding their current achievement level and the distance to their expected goals.

Discrepancies between expected and current performance can influence individual responses, leading to either increased effort or dissatisfaction and quitting, depending on their self-efficacy beliefs. More specifically, short-term, specific goals enhance self-efficacy by providing clear standards for progress, while goals of appropriate difficulty levels promote motivation and achievement. Goals that seem unattainable with current performance are also referred to as stretch goals (76). In neurorehabilitation, stretch goals may be more suitable at the team level rather than for individual goal setting (28). While self-set goals lead to higher self-efficacy and improved self-regulation (72), unrealistic goals may benefit from being reclassified as stretch goals, with feedback on subdividing them into manageable sub-goals (28). Additionally, goals focusing on skill learning are more effective in enhancing self-efficacy and self-regulation than those focused solely on performance, influencing motivation levels (72). Goal setting affects motivation, as individuals experience an initial sense of self-efficacy, which is substantiated as they observe their goal attainment process (77). Feedback plays a critical role in this process by providing information about current self-efficacy and goal attainment, helping individuals adjust their goals and enhance the acquisition of new skills (78).

#### Goal-oriented action linking (GOAL)

Motivation plays a critical role in rehabilitation, but its integration into health research raises concerns (79, 80). In order to identify motivational factors that account for maintaining goal pursuit, the Goal Oriented Action Linking (GOAL) architecture proposed by Ballard and colleagues (31) provides a comprehensive explanation of how the motivational value of a goal changes across three gradients. The [1] *distance gradient* refers to the amount of progress needed for goal pursuit and is divided into a *discrepancy perspective* and a *proximity perspective*. The former refers to motivation being higher when there is distance from the goal (a positive distance gradient). The latter, refers to motivation being higher when the person is closer to the goal (a negative distance gradient). The [2] *time gradient* represents the value changes in relation to the amount of time remaining to attain the goal. The [3] *rate of progress gradient* is the interaction between distance and time gradient, and reflects the motivational value changes according to the rate of progress required for goal achievement. It is further divided into three perspectives based on the relationship between motivation level and difficulty of the task at hand: *expectancy perspective* suggests that motivation is higher when goals are easier to achieve, *difficulty perspective* suggests that motivation is higher when goals are harder to achieve, and *achievability perspective* suggests that motivation is higher at moderate levels of difficulty.

Ballard and colleagues (31) tested the GOAL model with 294 healthy adults in three experimental studies by manipulating distance, time to goal, and goal type (approach vs. avoidance) using Hierarchical Bayesian modeling to identify factors relevant in accounting for the changes in motivation during goal pursuit. Of particular importance, the *time gradient* revealed that deadlines had a stronger motivational effect on approach goals than on avoidance goals. In avoidance goals, participants focused on avoiding immediate threats, often moving toward less immediate ones. Additionally, the results indicated that the type of goal (approach vs. avoidance) significantly impacted how rate of progress gradient influenced motivation.

These findings align with research on self-efficacy. People perceive difficult goals differently–some find them motivating, while others avoid them. Approach goals are generally more beneficial for health behavior change (81), but in cases of realistic fear, like avoiding a stroke, avoidance goals may be more effective (82). A recent study found that self-efficacy enhances the effectiveness of avoidance intentions by reducing anxiety, leading to better behavioral outcomes (82). Thus, boosting self-efficacy is crucial, especially when pursuing avoidance goals to prevent harmful outcomes.

Goals are fundamental in regulating human behavior, and in such cases, people must continually decide which goals to prioritize, how much effort to invest in achieving them, and when to shift focus to other goals (31). Motivation provides information on the direction and intensity based on the expectation and needs of the individual (83), reference points, or indicators of an individual’s current status toward achieving a goal, portray ways in which people conform or avoid particular goals (32). Research with stroke patients has shown that such patients want specific, consistent, and objective information regarding the purpose of their assessments and their recovery progress (84). Therefore, continuous feedback and referring back to their initial goals may also be important information that ought to be provided to such patients.

The reason behind whether they keep working towards a goal or give up may be impacted by individual differences (85). For example, individual differences, such as sensitivity to outcomes (approach vs. avoidance), optimism about goal achievement, tolerance for uncertainty, and perceptions of distance and time (31), contribute to shaping the motivation needed to achieve the goal. These variations influence the strength of individuals’ motivation and the strategies they employ in goal pursuit, making them important considerations for inclusion under personal factors in the ICF. In the following section, we will explore the potential application of the GOAL model in identifying key components to include in the ICF. Additionally, we will explain how self-efficacy would be an important component to be considered within personal factors.

### GOAL architecture, self-efficacy and personal factors to consider in the ICF

When taking in to consideration factors that may be incorporated in the personal factors within the ICF, the categorization of *thoughts and beliefs* proposed by Geyh and colleagues (14), under *subjective experience* may include aspects mentioned in the GOAL model and concepts of self-efficacy. Even though in their categorization, *distance*, *time*, and *rate of progress* of the GOAL model are not considered, we propose that these aspects are also of importance in understanding the thoughts and beliefs that represent “cognitive representations that are accessible to consciousness at a given point in time” (14) (p. 1734). Examples of *thoughts and beliefs* include personal memories, attitudes, attributions, expectations, and value and norms. This notion could be linked to the ICF category b1800 (experience of self, under body functions), but might be distinctly categorized to emphasize the distance/time/rate of progress gradient, while also accounting for individuals’ current, past, and future subjective experiences.

The GOAL architecture contributes to the understanding of how the willingness to exert effort changes depending on the amount and length of time that is available in achieving the goal (31). Research on the cost of effort has shown that people tend to choose tasks that require less effort (86). The time gradient is important in such cases as they provide more time to sample the value of the choice. When considering the relative values of choices at hand, attention is known to be an important component as it helps accumulate evidence by attending to options they believe have higher value (87), which is essentially what evidence accumulation models propose. Evidence accumulation models explain cognitive processes like information processing efficiency, response thresholds, and motor response timing (88). When evaluating goal-pursuit choices, considering the time gradient is crucial, as it reflects the duration needed for evidence accumulation.

In the evidence accumulation models, the necessary amount of information to make a decision is called the *decision threshold* (89). A higher threshold leads to slower but more accurate decisions, while a lower threshold results in quicker but less accurate choices. Deadlines influence threshold adjustments, with individuals lowering their thresholds or making random choices to meet time limits (87). Deadlines more strongly motivate those pursuing approach goals, while avoidance goals are driven by proximity to negative outcomes (31). When options are similar, balancing the value of additional information with time becomes crucial (90). Prior knowledge influences decision-making, as individuals tend to favor familiar choices, aligning with the first modulation assumption of the PSI model, where positive affect supports transitioning from habitual to intentional behavior (40, 91). Previous experience, if unaffected by brain damage, can affect decision thresholds (87).

When considering aspects to incorporate as personal factors in the ICF, it would be important to consider time and distance gradient together with the PSI, including the assessment of positive/negative affect, to decide whether they have enough time or not to process the necessary information in relation to the goals that they are pursuing, and identifying the current status of an individual and the distance to the desired status. Additionally, given the tendency for individuals to focus on one option over another when perceived values are similar, it is also crucial to account for previous experiences and habitual decision-making tendencies when evaluating personal factors in the ICF.

Individual differences in motivation are also influenced by people’s belief, or confidence, that serve as a “value signal” to decide whether engaging in a task is worthwhile. Confidence, much like rewards, helps people prioritize tasks: they tend to choose tasks where they feel more confident about succeeding (87). This preference for high-confidence tasks might also be linked to effort avoidance, where people choose easier tasks because they elicit greater confidence. People constantly evaluate the costs and benefits of tasks, using abstract features like their overall performance on a task (92). This information allows them to adjust their behavior flexibly, deciding what tasks to pursue or abandon based on the previously learned relative values of those tasks. Roualt and colleagues (93) found that individuals prefer tasks they feel confident in, regardless of difficulty, and this preference persists even after feedback. The findings of their study highlighted how immediate confidence and beliefs about one’s abilities interact, impacting decision-making and performance predictions, with implications for self-efficacy and depression.

Self-efficacy also enhances motor acquisition by increasing attention and motivation, and decreasing negative emotions such as anxiety (78, 94). Therefore, it might also be important to take in to consideration the aspect of self-efficacy as personal factors in the ICF. In the context of rehabilitation, improving balance and mobility is a central concern in stroke recovery interventions, with the execution of performance relying on “falls self-efficacy” and “balance self-efficacy,” denoting patients’ confidence levels in avoiding falls or performing activities without losing balance (95). Schmidt and her colleagues (96) found that balance and fall self-efficacy were strongly correlated with activity and participation in chronic stroke patients, with balance self-efficacy as the strongest predictor. They concluded that psychological factors like self-efficacy are critical in recovery. Therefore, we propose that falls and balance self-efficacy could be included as personal factors in the ICF for relevant populations. However, behavioral change is not influenced only by self-efficacy (97). Consideration of self-efficacy ought to be undertaken in conjunction with the value people perceive in relation to the goals that they are pursing (98). Moreover, incorporating elements from the GOAL model, such as the time gradient, can provide a more comprehensive understanding of the attention and information processing capacities that individuals can muster when pursuing their goals.

Since self-efficacy overlaps with important constructs such as perceived control, outcome expectations, perceived value of outcomes, attributions, and self-concept (77), it becomes important to align the definition with the measurement, as what is being assessed becomes ambiguous and may produce inconsistent results if defined differently (99). Self-efficacy is thought to be domain-specific (67), often assessed through questionnaires in rehabilitation studies. For example, the Stroke-Self Efficacy Scale was created by Jones and her colleagues (100), and it has been validated by comparing it to the “falls efficacy scale.” A systematic review of rehabilitation studies from 2015 to 2020 identified 80 different measures of self-regulation, with the General Self-Efficacy Scale being the most common (101). General self-efficacy, self-esteem, perceived behavioral control, and locus of control are frequently used interchangeably, contributing to confusion and the potential misuse of the self-efficacy definition in the rehabilitation field (102). Moreover, health-related research frequently conflates self-efficacy with motivation, highlighting the need to examine motivational factors in health behaviors (79). Given the domain-specific nature of self-efficacy, variations in measurement tools can complicate its inclusion as a personal factor in the ICF. Clarifying measurement objectives and using frameworks like the PSI and GOAL model could address these challenges.

## Summary and conclusions

In this theoretical paper, we have focused on the aspect of personal factors within the ICF which have not been categorized yet in the ICF. The ICF considers the environment and background information of individuals, including personal details, to assess individual needs, determine appropriate goals, and evaluate how these goals can be achieved. The strength of the ICF lies in its use as a common language to describe rehabilitation progress at various time points and among different specialists. This facilitates the establishment of a shared reporting system, helping to avoid ambiguities and inconsistencies (28). On the other hand, the weakness lies in the ICF not providing specific operationalization of the concepts involved and the absence of specific measurement tools (103). This is problematic because there is a tendency for a conceptual difference to occur between the internal view of patients about their health and the external views held by doctors and other professionals about the same health situation (104). Additionally, questions have arisen about the ICF’s ability to adequately reflect the subjective and social dimensions of disability (105). This includes the aspect of personal factors, as currently personal factors are not being categorized because of a lack of a standardized and comprehensive understanding of which personal factors are of interest within the ICF framework, and how those factors should be distinguished from other ICF categories (13).

**Figure 2:**
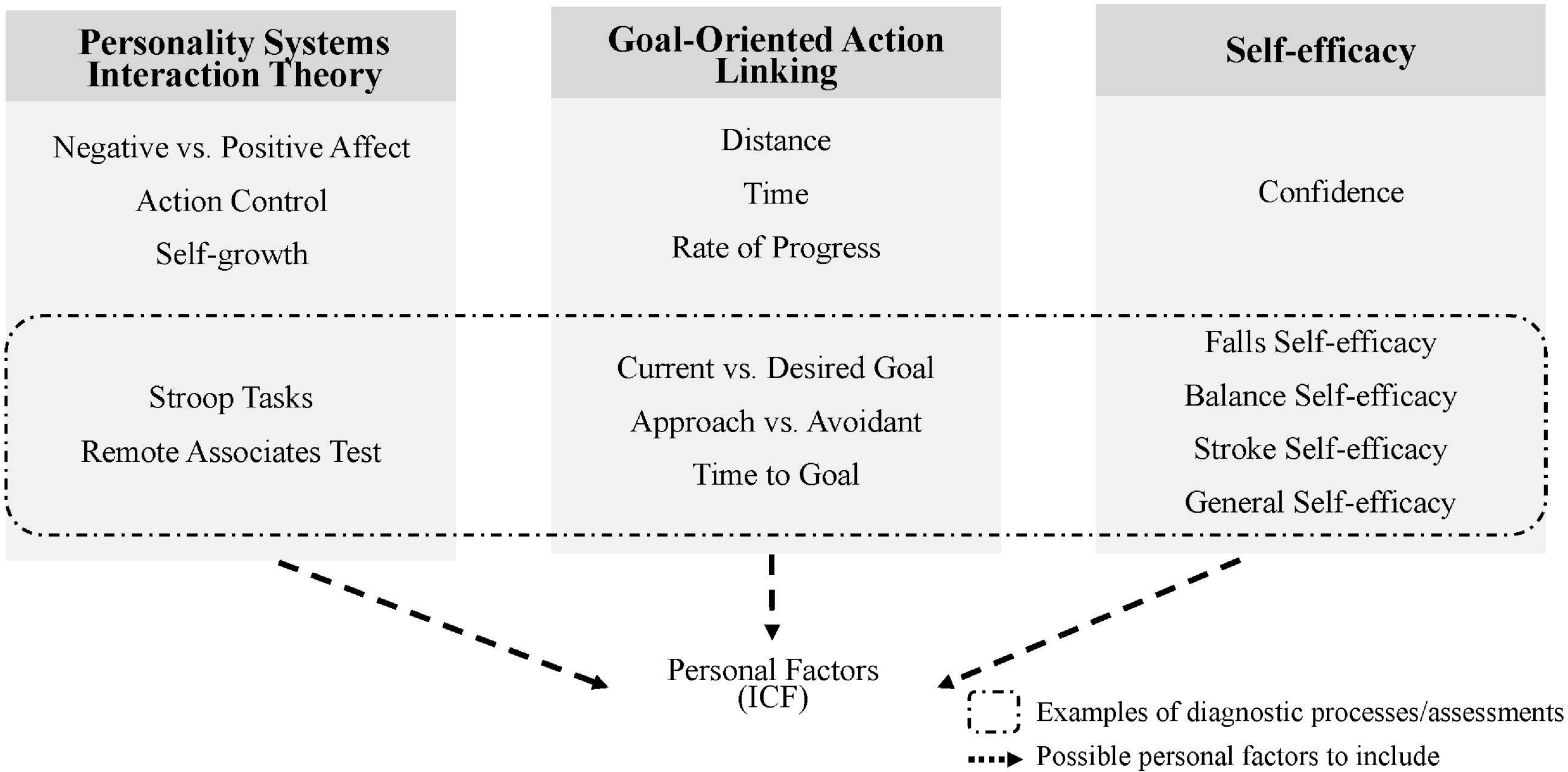
Potential personal factors that may be integrated into the ICF with its possible diagnostic and feedback interventions, incorporating the PSI Theory (30), the GOAL model (31), and Self-efficacy [e.g. (69)].

We have provided potential theoretical frameworks that may assist in identifying personal factors that may be relevant to the ICF (see Figure 2 for a summary). Because defining goals is an important aspect in rehabilitation (106–108), we focused on the role of goal setting in formulating relevant theoretical frameworks. The PSI theory (30) was explained with the objective of identifying personal variability involved in goal pursuit. We identified feelings such as negative and positive affect to be important personal factors to account for the extent to which individuals are able to face adverse situations, such as failure, and to control their habitual behaviors. For rehabilitation practitioners, understanding the emotions of each patient may enable them to offer tailored behavioral control strategies aligned with patients’ typical emotional reactions. For instance, if a patient exhibits strong responses to negative emotions, practitioners might recognize a tendency toward habitual behaviors and intervene with positive reinforcement, focusing on managing and redirecting these habitual responses effectively. Moreover, we have also been able to identify the distinction in the definition of motives under body functions and structures with that of personal factors, which might emphasize the aspect of emotions more than the mental functions involved in executing an action.

We have also suggested the use of the GOAL model (31) to understand how the motivational value of a goal changes across three gradients, *distance*, *time*, and *rate of progress*. Amongst the three, we have particularly focused on the importance of the *time gradient*, as time has been found to be important when individuals are in a situation where they have a decision to make and in need of assessing the value attached to achieving the goals. Moreover, the values they come up with seem to be related to their self-efficacy and the amount of effort they put into achieving the goals, which appear to be additional components important to consider as personal factors. However, assessment issues related to self-efficacy have been identified, and here, research in rehabilitation tends to confound the meaning of self-efficacy with controllability (109) and motivation (79). As self-efficacy is known to be domain-specific, it would be necessary to clarify what needs to be measured, the objectives of using any particular questionnaire, and basing the choices on frameworks such as the PSI and the GOAL model to solve this issue.

Personal factors may also be relevant in active engagement in daily activities for patients beyond the rehabilitation context. According to Winstein and Kay (110), “consideration of an individual’s fundamental psychological needs for competence, autonomy, and social relatedness within the framework of rehabilitation therapy is an effective way to employ intrinsic motivation and positively benefit motor learning and recovery” (p. 346). Because the goal of rehabilitation not only focuses on recovering body functions but also on contributing to the patients’ autonomy and quality of life (111), it would be important to also consider social roles that may affect these variables so the patients can actively engage in achieving their goals even after their discharge from hospital care. Stroke patients undergoing rehabilitation are required to be active participants in their rehabilitation practices, and motivation is crucial for active engagement (112). To enable better support for the patients for their continuing effort in achieving their goals outside of rehabilitation context, it would be essential for rehabilitation practitioners to understand their personal and contextual factors and actively engage them in establishing goals, employ strategies for goal achievement, and monitor their progress, leveraging their self-efficacy (99). In this context, psychological factors such as PSI, the GOAL model, and self-efficacy, as summarized in Figure 2 represent key components of personal factors that should be considered throughout rehabilitation practices and beyond to effectively promote social participation.

## Limitations and future directions

This study aimed to identify theoretical frameworks that could be considered in determining appropriate personal factors to include within the ICF. We conducted a conceptual literature review to match the exploratory nature of that aim. While potential selection bias cannot be fully ruled out, the focus of this study was on exploring and proposing theoretical frameworks relevant to conceptualizing personal factors, rather than on conducting a comprehensive evaluation of specific studies or intervention effectiveness.

We suggested the PSI theory and GOAL model, both of which have primarily been studied with healthy individuals. To gain a clearer understanding of their applicability in real-world rehabilitation settings, these theories should be evaluated in stroke patients across different phases of recovery.

While this paper has primarily focused on stroke patients requiring multidisciplinary care, we believe that the PSI and GOAL models are equally applicable to outpatients and individuals with other health conditions, as they are based on fundamental human mechanisms that assume commonalities across cultures, behaviors, and minds (113). However, the debate on the existence of universal human traits persists, and it has been suggested that brain data may help uncover fundamental cognitive structures that in turn could help resolve this issue (27). We propose that future studies test the PSI and GOAL models cross-culturally and across various health conditions, incorporating brain data to assess their applicability across cultures. Additionally, these models should be examined in different age groups, using the International Classification of Functioning, Disability, and Health for Children and Youth (ICF-CY), which addresses development and age-specific factors.
